# Detecting Interactive Gene Groups for Single-Cell RNA-Seq Data Based on Co-Expression Network Analysis and Subgraph Learning

**DOI:** 10.3390/cells9091938

**Published:** 2020-08-21

**Authors:** Xiucai Ye, Weihang Zhang, Yasunori Futamura, Tetsuya Sakurai

**Affiliations:** Department of Computer Science, University of Tsukuba, Tsukuba 3058577, Japan; s2020623@s.tsukuba.ac.jp (W.Z.); futamura@cs.tsukuba.ac.jp (Y.F.); sakurai@cs.tsukuba.ac.jp (T.S.)

**Keywords:** single-cell RNA-seq, machine learning, interactive gene groups, co-expression networks, subgraph learning

## Abstract

High-throughput sequencing technologies have enabled the generation of single-cell RNA-seq (scRNA-seq) data, which explore both genetic heterogeneity and phenotypic variation between cells. Some methods have been proposed to detect the related genes causing cell-to-cell variability for understanding tumor heterogeneity. However, most existing methods detect the related genes separately, without considering gene interactions. In this paper, we proposed a novel learning framework to detect the interactive gene groups for scRNA-seq data based on co-expression network analysis and subgraph learning. We first utilized spectral clustering to identify the subpopulations of cells. For each cell subpopulation, the differentially expressed genes were then selected to construct a gene co-expression network. Finally, the interactive gene groups were detected by learning the dense subgraphs embedded in the gene co-expression networks. We applied the proposed learning framework on a real cancer scRNA-seq dataset to detect interactive gene groups of different cancer subtypes. Systematic gene ontology enrichment analysis was performed to examine the detected genes groups by summarizing the key biological processes and pathways. Our analysis shows that different subtypes exhibit distinct gene co-expression networks and interactive gene groups with different functional enrichment. The interactive genes are expected to yield important references for understanding tumor heterogeneity.

## 1. Introduction

Recent advances in Next-generation sequencing (NGS) technologies have enabled the generation of high-throughput single-cell gene expression data exploring both genetic heterogeneity and phenotypic variation between cells [1,2]. Single-cell RNA-seq (scRNA-seq) acquires transcriptomic information from individual cells, providing a higher resolution of cellular differences and a better understanding of cell functions at genetic and cellular levels [3]. In contrast with traditional bulk RNA-seq that reveals the average gene expression of a collection of cells, scRNA-seq will allow researchers to uncover new and potentially unexpected biological discoveries [4]. scRNA-seq has been utilized to study cancer, where tumor heterogeneity poses significant challenges in the clinical diagnosis, cancer treatment, and patient survival [5]. The unprecedented ability of measuring gene expression from individual cells holds enormous potential for detecting the clinically important tumor subpopulations and understanding tumor heterogeneity [6].

Many machine learning methods have been applied to analyze scRNA-seq data for determining cell types and predicting diagnoses [7]. scRNA-seq data often comes with high dimensionality, which involves a large number of genes but a small number of samples. Since the limited number of samples may lead to overfitting due to the noisy genes [8], dimensionality reduction methods are usually carried out after counting normalization to avoid the curse of dimensionality, meanwhile provide visual representations of the cellular composition within high-dimensional data. Principal component analysis (PCA) [9] and t-Distributed Stochastic Neighbor Embedding (t-SNE) [10] are wildly used to project high-dimensional gene expression data into a low-dimensional space. Recently, uniform manifold approximation and projection (UMAP) [11] has been applied to visualize scRNA-seq data and shows better performance than t-SNE [12]. Other dimensionality reduction methods such as feature selection [13,14] can also be applied to delete the noisy genes and identify the most discriminant gene subset for distinguishing different types of cells and finding the biological information embedded in scRNA-seq data. Recently, some feature extraction tools have been developed for DNA, RNA and protein sequence analysis, such as BioSeq-Analysis [15] and BioSeq-Analysis2.0 [16]

Besides dimensionality reduction methods, clustering methods are critical to single-cell analysis, helping to understand potential cellular mechanisms [17]. Classic clustering methods such as K-means [18], hierarchical clustering [19], and EM [20] can be applied in single-cell clustering directly. Spectral clustering methods [21,22] which make use of the spectrum of graph Laplacian to reveal the cluster structure have been reported to be more effective than other classic clustering methods for scRNA-seq data [3]. Meanwhile, several analysis tools designed for scRNA-seq data provide clustering methods to improve the capability of data analysis, including Seurat [23], SINCERA [24], CIDR [25], SC3 [26], and SNN-cliq [27]. Based on the clustering results, diverse downstream expression analysis can be carried out, such as identification of subpopulations of cells and detection of differential expression signatures [28].

Existing methods have been proposed to detect the related genes causing cell-to-cell variability for studying gene expression dynamics [29,30]. Gene co-expression networks are a potent approach to the identification of genes not yet associated with explicit biological questions and for accelerating the interpretation of molecular mechanisms at the root of significant biological processes [31]. Some methods using gene co-expression networks have been proposed to identify important genes that are related to different cancer subtypes [32]. However, most existing methods detect the related genes separately, without considering gene interactions. Many human diseases are multigenic, which are caused by the mutations in multiple genes that all affect a single phenotypic trait [33]. Genes regulate the activity of one-another in large co-expression networks. Therefore, SNPs may not only affect the activity of a single target gene, but the activity of multiple biologically related genes within the same co-expression network to influence the manifestation of a phenotype [34]. Thus, it is necessary to detect interactive genes that are related to different cell subpopulations.

In this paper, to detect the interactive genes for scRNA-seq data, we proposed a novel learning framework based on co-expression network analysis and subgraph learning. Firstly, spectral clustering was utilized to identify the subpopulations of cells. Then, for each cell subpopulation, the genes more strongly differentially expressed were selected to construct a gene co-expression network. The topological overlap matrix was used to represent the gene connectivity. Finally, the interactive gene groups were detected by learning the dense subgraphs embedded in the gene co-expression networks. The proposed framework was applied on a real cancer scRNA-seq dataset to detect the interactive gene groups of different cancer subtypes. We performed systematic gene ontology enrichment analysis to examine the potential functions of the detected interactive gene groups by summarizing the key biological processes and pathways.

## 2. Materials and Methods

**Figure 1:**
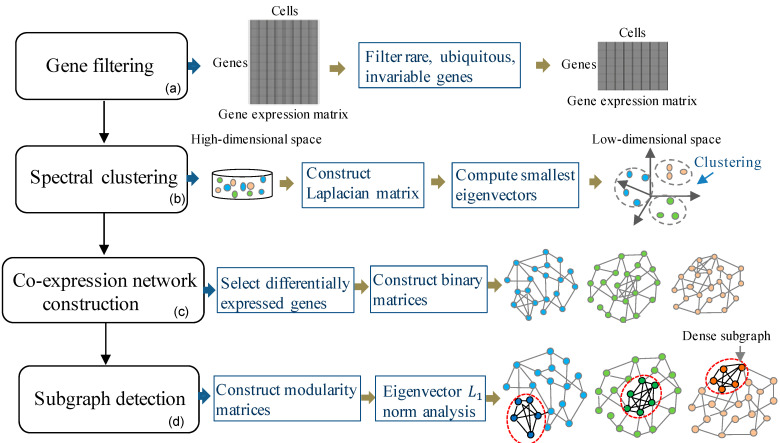
The proposed learning framework to detect interactive gene groups. Four major steps: (**a**) Filtering rare, ubiquitous, and invariable genes; (**b**) Spectral clustering to identify cell subpopulations; (**c**) Constructing gene co-expression networks; (**d**) Detecting dense subgraphs embedded in the gene co-expression networks.

An overview of the proposed learning framework is illustrated in Figure 1. The proposed framework mainly contains four stages. (a) Filtering rare, ubiquitous, and invariable genes. (b) Spectral clustering to identify cell subpopulations. (c) Constructing gene co-expression network for each cell subpopulation. (d) Detecting dense subgraphs embedded in the gene co-expression networks.

### 2.1. Methods: The Proposed Learning Framework

#### 2.1.1. Gene Filtering

In the gene filtering step, we filtered out the rare, ubiquitous, and invariable genes to focus on the intrinsic transcriptomic signatures of cells in the scRNA-seq data. Since the rare and ubiquitous genes are usually not useful for identifying different cell subpopulations, the genes that are expressed in less than r% of cells (i.e., rare genes) or expressed in at least (100−r)% of cells (i.e., ubiquitous genes) were firstly filtered out. We set r% as 6, as that considered in the previous study [35]. Then, the most c% variable gene set across the single-cells was identified by controlling the relationship between mean expression and variability.

#### 2.1.2. Spectral Clustering to Identify Cell Subpopulations

Given a set of *n* data samples $X=\{x_{1},x_{2},...,x_{n}\}$ in $R^{d}$, the objective of spectral clustering is to divide the data samples into *K* clusters. Spectral clustering consists of two main steps: (1) Dimensionality reduction based on the eigenvectors of the Laplacian matrix; (2) Finding clusters in the low-dimensional space.

In the first step, a similarity matrix was constructed to calculate the Laplacian matrix. The similarity matrix *S* has pairwise similarities $s_{ij}$ (i,j=1,...,n) as its entries, i.e., $S=(s_{ij})$. By using the Gaussian kernel function, the pairwise similarity is calculated as

(1) $$s_{ij}=\left\{\begin{array}{cc} \exp(-\frac{∥x_{i}-x_{j}{∥}^{2}}{2\sigma^{2}}), & \mathrm{if}\;i\neq j,\\ 0, & \mathrm{if}\;i=j, \end{array}\right.$$

where $∥x_{i}-x_{j}∥$ is the Euclidean distance between data samples $x_{i}$ and $x_{j}$, σ is the kernel parameter. Furthermore, the undirected *k*NN graph was applied to sparse the similarity matrix, by which $s_{ij}$ is calculated as Equation (1) when $x_{i}$ is one of the *k* nearest neighbors of $x_{j}$ or $x_{j}$ is one of the *k* nearest neighbors of $x_{i}$, otherwise $s_{ij}=0$. The normalized Laplacian matrix *L* was then calculated as L=D−S, where D is a n×n diagonal matrix with $d_{i}=\sum_{j=1}^{n}s_{ij}$ on the diagonal. The *K* smallest eigenvectors corresponding to the *K* smallest eigenvalues of *L* were computed to form a low *K*-dimensional space.

In the second step, the *K*-means clustering method was performed to divide the data into *K* clusters in the low-dimensional space. By using spectral clustering, the cells in a cluster were identified as the same subpopulation.

#### 2.1.3. Differentially Expressed Gene Selection

Genes that are more strongly differentially expressed (DE) are more likely to cause separated clusters of cells [36]. We selected the DE genes for the samples in each cluster to construct the gene co-expression networks.

To select the DE genes, we used the Welch *t*-test [37] to test differences in expression between clusters. Pairwise comparisons between clusters were performed for each gene. The genes differentially expressed in any pairwise comparison between clusters will be given a low *p*-value. For a cluster, we combined the *p*-value for each gene by combining the *p*-values across the pairwise comparisons involving this cluster [38]. For example, considering 3 clusters and combining the *p*-value of each gene for cluster 1. Pairwise comparisons between clusters 1 and 2, and between clusters 1 and 3 were performed for each gene, respectively. Then the *p*-value of each gene in the two comparisons were combined. The combined p-value for each gene was calculated as the middle-most value by applying the Holm-Bonferroni correction [39] across its *p*-values. Thus, in each cluster, a gene will achieve a low combined *p*-value if it is strongly differentially expressed in all pairwise comparisons to other clusters.

Then, we calculated the false discovery rate (FDR) by the Benjamini–Hochberg method [40] based on the combined *p*-value. For each cell subpopulation, the genes with 5% FDR were selected as the DE genes.

#### 2.1.4. Gene Co-Expression Network Construction

For each cell subpopulation, we constructed a gene co-expression network based on the selected DE genes. A gene co-expression network is a transcript–transcript association network, generally reported as an undirected graph, in which genes are connected when there is a significant co-expression relationship between them [41].

Firstly, we calculated the adjacency matrix $A=(a_{ij})$, in which $a_{ij}$ is the adjacency value between gene *i* and gene *j*. $a_{ij}$ is calculated as

(2) $$a_{ij}={\left|w_{ij}\right|}^{\beta },$$

where $w_{ij}$ is the gene-wise similarity which is calculated as the absolute value of the pairwise Pearson correlation between gene *i* and gene *j*, β is the single soft threshold which is chosen by scare free topology criterion.

Then, we applied the topological overlap matrix (TOM) to calculate the gene connectivity in the co-expression network. TOM provides the implication of the connected genes and their useful biological function or pathway [42]. The entries of TOM, i.e., $t_{ij}$, is calculated based on $a_{ij}$ as follows.

(3) $$t_{ij}=\frac{\sum_{m}a_{im}a_{mj}+a_{ij}}{\min\left\{\sum_{m}a_{im},\sum_{m}a_{mj}\right\}+1-a_{ij}}.$$

We further constructed a binary matrix *B* by using 1 to represent the strong similarity and using 0 to represent the weak similarity. The top g% values in TOM were set as 1 and the rest were set as 0. The binary matrix $B=(b_{ij})$ directly presents the connectivity between genes in the co-expression network. That is, $b_{ij}=1$ if there is an edge between gene *i* and gene *j* and $b_{ij}=0$ otherwise. In this paper, the g% was set by experience.

#### 2.1.5. Subgraph Detection

We detected the dense subgraphs embedded in the gene co-expression network based on eigenvector $L_{1}$ norms of a modularity matrix [43,44]. Newman’s notion of the modularity matrix [45] associated with an unweighted, undirected graph is given by

(4) $$M=B-\frac{1}{2|E|}HH^{T}.$$

Here *B* is the binary matrix used to construct the gene co-expression network. *H* is the degree vector of the co-expression network, where the *i*th component of *H* is the number of edges adjacent to gene *i*. |E| is the total number of edges in the co-expression network. Since *M* is real and symmetric, it admits the eigendecomposition $M=U\Lambda U^{T}$, where *U* is a matrix with each column being an eigenvector of *M*, and Λ is a diagonal matrix of eigenvalues.

We detected the dense subgraphs based on $L_{1}$ properties of the largest eigenvectors corresponding to the largest eigenvalues of the modularity matrix. The $L_{1}$ norms of an eigenvector $v_{i}={\left[v_{i1},v_{i2},...,v_{iz}\right]}^{T}$ is calculated as

(5) $$\|v_{i}\|=\sum_{j=1}^{Z}\left|v_{ij}\right|.$$

Here *Z* is the number of genes in the co-expression network. The $L_{1}$ properties of the largest eigenvectors have been exploited in a graph-theoretic setting for finding maximal cliques. If a small set of genes are interactive, i.e., forms a community group in the co-expression network, there will be an eigenvector well aligned with this set, which implies that the $L_{1}$ norm of this eigenvector would be smaller than that of an eigenvector with a similar eigenvalue when there is no dense subgraph. The genes involved in dense subgraph are probably interactive since they are tightly connected.

### 2.2. Materials

In this study, we used a single-cell expression dataset from a recent scRNA-seq study, i.e., GSE72056 [46], which was selected from the data repository NCBI Gene Expression Omnibus. In this dataset, 4645 single cells with 23684 genes were isolated from 19 patients with melanoma tumor. There are 1257 malignant melanoma tumor cells and 3388 benign tumor cells. The detailed number of cells in each sample/patient is listed in Table 1.

The proposed framework was applied to the malignant melanoma tumor cells to identify the cancer subtypes and detect the interactive gene group in each cancer subtype. As shown in Table 1, the 1257 malignant cells were derived from 15 patients. The dataset was transformed by logTPM before being processed by the proposed framework.

## 3. Results

### 3.1. Identification of Cancer Subtypes

**Figure 2:**
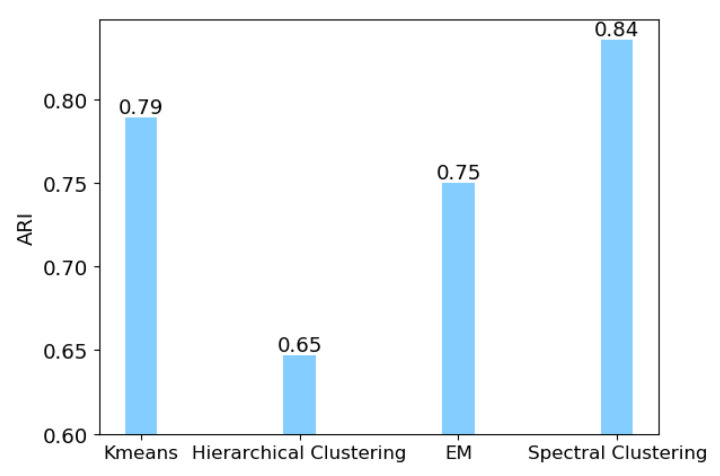
Performance comparison of different clustering methods. Adjusted rand index (ARI) is employed to measure the accuracy of clustering results.

We applied spectral clustering to identify the cancer subtypes due to its superior performance compared to other classic clustering methods. To evaluate the performance of spectral clustering, we first compared spectral clustering with other classic clustering methods, i.e., K-means [18], hierarchical clustering [19], EM [20], on the clustering task: clustering the 4645 cells in GSE72056 into two clusters (malignant and benign tumor cells). We used the adjusted rand index (ARI) [47] to measure the accuracy of clustering results. A larger value of ARI indicates a better clustering result. The comparison result is shown in Figure 2. We can see that spectral clustering outperforms other classic clustering methods.

**Figure 3:**
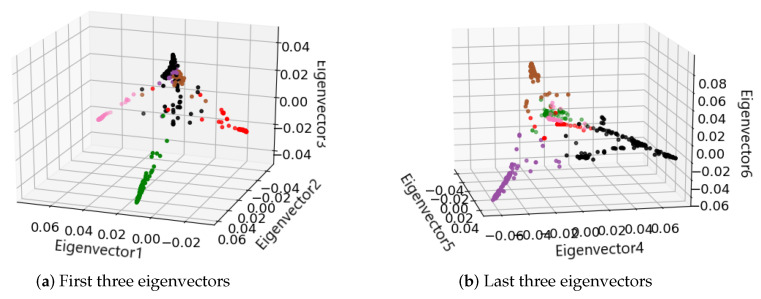
Three-dimensional spaces constructed by (**a**) the first three eigenvectors and (**b**) the last three eigenvectors. Different colors denote different clusters output by spectral clustering.

Then, spectral clustering was applied to identify the cancer subtypes of malignant melanoma tumor in GSE72056. Since there is no ground truth of the clusters for these malignant cells, we applied Calinski-Harabaz Index [35] to decide the number of clusters. Spectral clustering identified six clusters, i.e., six cancer subtypes, in the dataset. In spectral clustering, the six smallest eigenvectors corresponding to the six smallest eigenvalues of the Laplacian matrix were computed to form a low six-dimensional space. Since six-dimensional space cannot be visualized directly, we show the three-dimensional spaces constructed by the first three eigenvectors and the last three eigenvectors in Figure 3a,b, respectively. We can see that the three clusters denoted by red, pink, and green colors can be separated by the first three eigenvectors, and the other three clusters denoted by brown, purple, and black colors can be separated by the last three eigenvectors. Thus, by using the six eigenvectors, the six clusters can be separated and identified.

**Figure 4:**
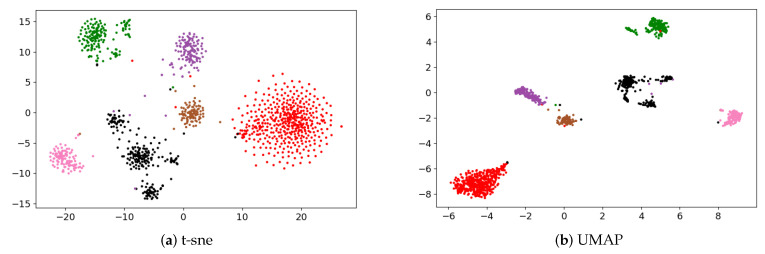
Visualization of cancer subtypes identified by spectral clustering from human melanoma scRNA-seq data set in two-dimensional space constructed by (**a**) t-SNE and (**b**) UMAP, respectively. Different colors denote different clusters output by spectral clustering.

We also visualized the clustering result of spectral clustering by t-SNE and UMAP, the corresponding results are shown in Figure 4a,b, respectively. Spectral clustering displays six clearly recognizable clusters in the two-dimensional space constructed by both t-SNE and UMAP. The two-dimensional space constructed by UMAP shows more clearly recognizable clusters than that by t-SNE.

We listed the 6 cell subpopulations presented in each sample/patient in Table 2. For each sample/patient, the majority of cells belonging to a subtype was highlighted in bold-face type. We can see from Table 2, in most cases, the subpopulations are indeed present in the same patients (100%) or the majority of cells in the same patients (>92%). Some patients may refer to more than one melanoma subtype, such as Melanoma_60 and Melanoma_94.

### 3.2. Detecting Interactive Gene Groups

We detected the interactive gene groups from each cancer subtype identified by spectral clustering. In each cancer subtype, the DE genes were selected to construct a gene co-expression network. The numbers of selected DE genes for subtypes 1 to 6 are 3092, 4679, 5644, 4364, 4538, and 2533, respectively. We used the WGCNA [48] to calculate the TOM matrix. The top g% values to be 1 for subtypes 1 to 6 were set as 3.5, 1.5, 0.4, 0.9, 0.9, and 0.9, respectively. A binary matrix was formed based on the TOM matrix to decide the edges in the gene co-expression network. Then, the interactive gene groups were detected based on the eigenvector $L_{1}$ norms of the modularity matrix which was calculated based on the binary matrix.

**Figure 5:**
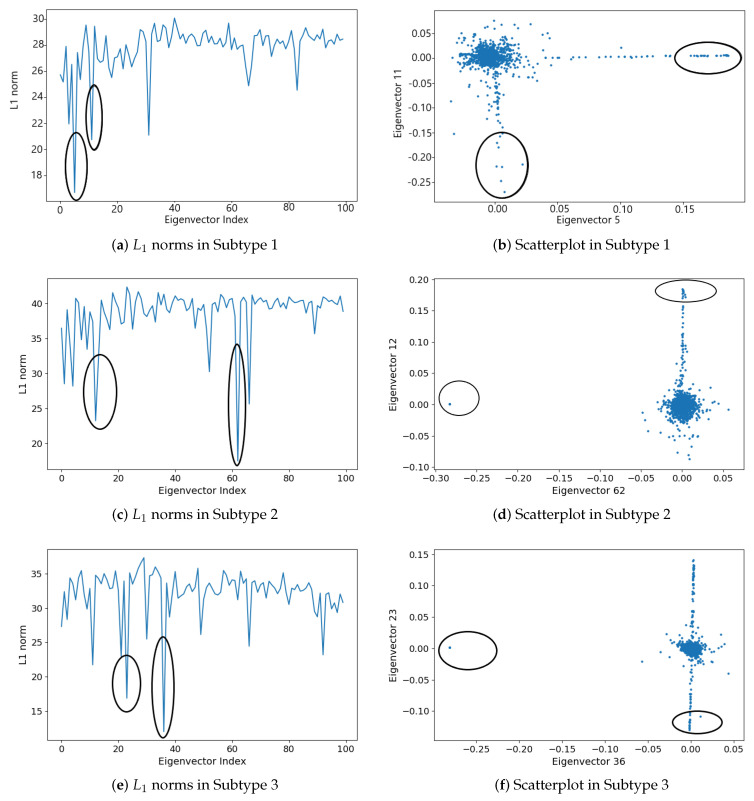
Eigenvector $L_{1}$ norms (left column): (**a**) $L_{1}$ norms in Subtype 1, (**c**) $L_{1}$ norms in Subtype 2, and (**e**) $L_{1}$ norms in Subtype 3. Scatterplots of the projection into the subspace defined by the indicated eigenvectors (right column): (**b**) Scatterplot in Subtype 1, (**d**) Scatterplot in Subtype 2, and (**f**) Scatterplot in Subtype 3.

**Figure 6:**
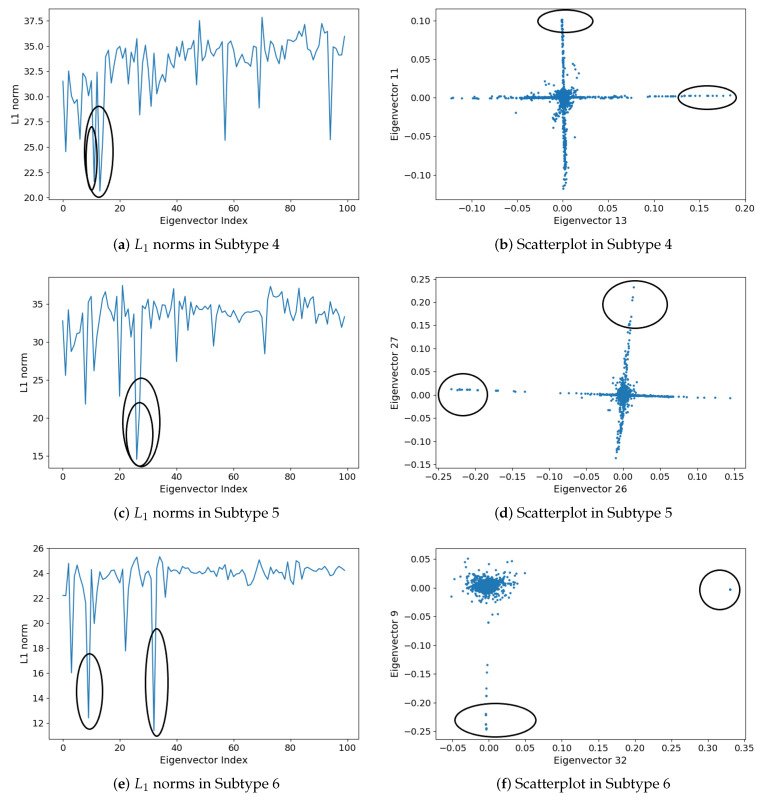
Eigenvector $L_{1}$ norms (left column): (**a**) $L_{1}$ norms in Subtype 4, (**c**) $L_{1}$ norms in Subtype 5, and (**e**) $L_{1}$ norms in Subtype 6. Scatterplots of the projection into the subspace defined by the indicated eigenvectors (right column): (**b**) Scatterplot in Subtype 4, (**d**) Scatterplot in Subtype 5, and (**f**) Scatterplot in Subtype 6.

For each cancer subtype, we computed the largest 100 eigenvectors of the modularity matrix and the $L_{1}$ norm of each eigenvector. Comparing each $L_{1}$ sequence to a “smoothed” version, we selected the two eigenvectors that deviate the most from this trend [43]. For the six cancer subtypes, the two eigenvectors that deviate most are those with the smallest $L_{1}$ norm. Figure 5 and Figure 6 show the plots of the $L_{1}$ norms of the largest 100 eigenvectors and the scatterplots in the space of the corresponding two eigenvectors with the smallest $L_{1}$ norm. The eigenvectors declared are highlighted by circles.

The dense subgraphs detected by $L_{1}$ analysis are presented in Table 3. Two subgraphs are first chosen from each cancer subtype, corresponding to the points highlighted by circles in the scatterplots in Figure 5 and Figure 6. The two subgraphs are denoted as Subg 1 and Subg 2 in Table 3. For each subgraph, we listed the size (number of genes), density (internal edges divided by the maximum number of edges), and the eigenvector that separates it from the co-expression network. $e_{j}$ denotes the *j*th largest eigenvector. We can see from Table 3, the detected subgraphs are quite dense, all with 100% density. That is, the genes are connected to each other in each detected subgraph.

**Figure 7:**
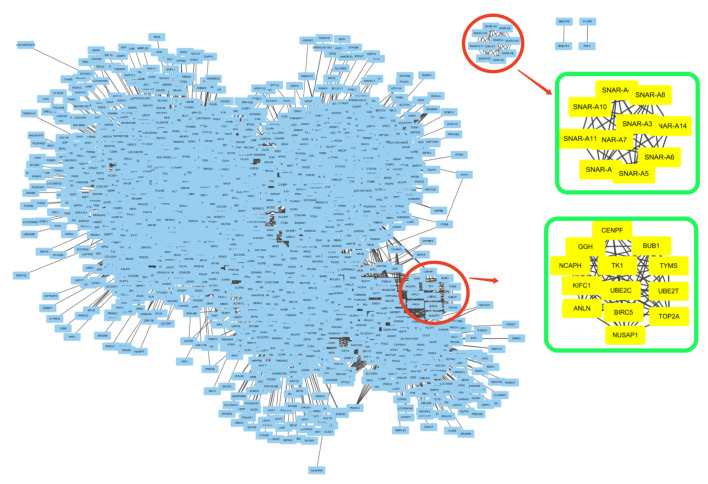
Two detected subgraphs in the gene co-expression network of cancer subtype 2. Two detected subgraphs are highlighted by red circles. Genes in the subgraphs are shown in the green squares.

We then examined the genes in the detected dense subgraph. We found that the genes in a detected subgraph are highly connected, however, they may isolate from other genes outside the subgraph. For example, subgraph 1 detected in cancer subtype 2, as shown in Figure 7. Figure 7 shows the gene co-expression network of cancer subtype 2, constructed by the Cytoscape software [49]. Two detected subgraphs are highlighted by red circles in the gene co-expression network. To see the genes in the subgraphs, we further enlarge the two detected subgraphs and show them in two green squares, respectively. The above green square shows Subg 1, in which the 10 genes, i.e., SNAR-A9, SNAR-A6, SNAR-A14, SNAR-A11, SNAR-A4, SNAR-A8, SNAR-A7, SNAR-A3, SNAR-A5, SNAR-A10, are highly connected. These genes are not connected to other genes outside Subg 1. The genes in Subg 1 have a very close relationship since they have the same prefix name. Note that the scatterplots of these genes in the space of two eigenvectors in Figure 5d, i.e., the points in the left circle, also isolate from other points/genes. Similar subgraphs are detected in subtypes 3 (Subg 1) and 6 (Subg 1), which are corresponding to the points in the left circle in Figure 5f and the points in the right circle in Figure 6f, respectively. The genes in Subg 1 of subtypes 3 are CT47A4, CT47A10, CT47A6, CT47A2, CT47A3, CT47A5, CT47A11, CT47A12, CT47A8, CT47A9, CT47A1, CT47A7, while the genes in Subg 1 of subtypes 6 are SNAR-A6, SNAR-A5, SNAR-A4, SNAR-A7, SNAR-A9, SNAR-A10, SNAR-A3, SNAR-A8. These genes in the detected subgraph all have the same prefix name.

We further performed systematic gene ontology enrichment analysis on the genes in a subgraph by using DAVID tools and summarize the key biological processes and pathways [50]. We have detected two subgraphs for each cancer subtype. Table 4 lists the enrichment analysis of the genes in one subgraph for each cancer subtype, in which the genes in the listed subgraph that are more enriched than those in another. For example, the genes in Subg 1 for cancer subtype 1 are listed since the analysis of genes in Subg 1 are more enriched than those in Subg 2. These modules are enriched for biologically important processes that are relevant to melanoma, such as cell cycle. The abnormal proliferation resulting from alterations in cell cycle regulatory mechanisms will lead to the transformation of melanocytes to melanoma cells [51]. In Table 4, we also summarize the number of genes that are involved in the same term type and write it in brackets, e.g., BP: cell cycle (18) means that 18 genes in Subg 1 are involved in BP: cell cycle, which are BUB1, FANCI, TPX2, ASPM, ANLN, AURKB, BIRC5, CENPF, CENPM, CDK1, DTL, MKI67, NUSAP1, PKMYT1, TYMS, TOP2A, UBE2C, UHRF1. In subtype 2, the largest number of genes belonging to the same type is 10, which are BUB1, ANLN, BIRC5, CENPF, KIFC1, NCAPH, NUSAP1, TYMS, TOP2A, UBE2C, and they are involved in BP: mitotic cell cycle process. In subtype 3, the most connected genes are also involved in BP: mitotic cell cycle process., which are NDC80, RACGAP1, TTK, CCNB1, CDKN3, CKAP2, FAM64A, FOXM1, KIF14, KIF20A, KIF20B, KIF4A, SKA3. We can see that these genes are different from those involved in BP: mitotic cell cycle process in subtype 2. A similar result can be found in the most highly connected genes in subtype 4 and subtype 6, which are involved in the same term type but the related genes are different. That may be because for different cancer subtypes the informative genes are different. We also can see from the enrichment analysis results in Table 4, the genes in the detected subgraph are closely related. For example, there are 20 genes in Sunb1 of subtype 1 and 18 of them are involved in the same term type. For Subg 2 in subtype 5, all the genes are involved in the same term type, i.e., BP: cellular macromolecule. The interactive genes detected in different cancer types are expected to yield important references for finding new markers and understanding tumor heterogeneity.

## 4. Conclusions and Discussion

scRNA-seq brings unprecedented insights into cellular heterogeneity, in which detecting the related genes causing cell-to-cell variability is critical. The related genes are usually detected separately without considering gene interactions. However, considering gene interaction is important since many human diseases are multigenic. In this paper, we proposed a novel learning framework to detect the interactive genes for scRNA-seq data based on co-expression network analysis and subgraph learning. We identified the cell subpopulations using spectral clustering and selected the differentially expressed genes to construct a gene co-expression network for each cell subpopulation. The interactive gene groups were detected by learning the dense subgraphs embedded in the gene co-expression networks. We applied the proposed learning framework on the real melanoma tumor scRNA-seq dataset. Six cancer subtypes were identified, and we detected the interactive gene groups from each cancer subtype. The genes were highly connected, i.e., connected to each other, in each detected gene group. Systematic gene ontology enrichment analysis was performed to examine the potential functions of the detected interactive genes by summarizing the key biological processes and pathways. Our analysis shows that different subtypes exhibit distinct gene co-expression networks and interactive gene groups with different functional enrichment. The interactive genes are expected to yield important references for understanding tumor heterogeneity.

Although our framework is proposed for scRNA-seq data and the experimental results are from the application on melanoma tumor dataset, the proposed framework is generally applicable to other types of biological data and other types of tumors. For example, the proposed framework can be apply to protein datasets to analyze the signal transduction pathways in protein interaction networks. Other types of biological data with the need for detecting interactive groups can apply the subgraph detection methods in the proposed framework.

Nevertheless, the current learning models in the proposed framework still have some limitations. Firstly, we used a published dataset with a low number of cells. In future work, we will analyze scRNA-seq datasets with a larger number of cells to better demonstrate the power of the proposed framework. Secondly, some known human gene-disease interactions can be integrated to improve the learning models. For example, using the known information to improve the model parameter setting. Thirdly, some ensemble learning and feature selection procedures can be properly integrated into the clustering process to enhance performance. Fourthly, significance of the findings can be much more articulate and interpreted in the light of the up-to-date knowledge of melanoma biology. We will leave these issues for future work.

## Figures and Tables

**Table 1:** Number of cells in each sample/patient.

Sample ID	Total Cells	Benign Cells (Percentage)	Malignant Cells (Percentage)
Melanoma_53	143	127 (88.8%)	16 (11.2%)
Melanoma_58	142	142 (100%)	0
Melanoma_59	70	16 (22.9%)	54 (77.1%)
Melanoma_60	226	217 (96.0%)	9 (4.0%)
Melanoma_65	63	59 (93.7%)	4 (6.3%)
Melanoma_67	95	95 (100%)	0
Melanoma_71	89	35 (39.3%)	54 (60.7%)
Melanoma_72	181	181 (100%)	0
Melanoma_74	147	147 (100%)	0
Melanoma_75	344	341 (99.1%)	3 (0.9%)
Melanoma_78	131	11 (8.4%)	120 (91.6%)
Melanoma_79	896	428 (47.8%)	468 (52.2%)
Melanoma_80	480	355 (74.0%)	125 (26.0%)
Melanoma_81	205	72 (35.1%)	133 (64.9%)
Melanoma_82	84	52 (61.9%)	32 (38.1%)
Melanoma_84	159	145 (91.2%)	14 (8.8%)
Melanoma_88	351	234 (66.7%)	117 (33.3%)
Melanoma_89	475	377 (79.4%)	98 (20.6%)
Melanoma_94	364	354 (97.3%)	10 (2.7%)

**Table 2:** Cell subpopulations presented in each sample/patient. The majority of cells belonging to a subtype was highlighted in bold-face type.

Sample ID	Subtype 1	Subtype 2	Subtype 3	Subtype 4	Subtype 5	Subtype 6
Melanoma_53	0	0	**16 (100%)**	0	0	0
Melanoma_59	**52 (96.3%)**	0	0	0	0	2 (3.7%)
Melanoma_60	1 (11.1%)	0	0	**6 (66.7%)**	1 (11.1%)	1 (11.1%)
Melanoma_65	**4 (100%)**	0	0	0	0	0
Melanoma_71	**50 (92.6%)**	1 (1.8%)	0	0	0	3 (5.6%)
Melanoma_75	3 (100%)	0	0	0	0	0
Melanoma_78	6 (5.0%)	0	0	**114 (95.0%)**	0	0
Melanoma_79	2 (0.4%)	**465 (99.4%)**	0	0	1 (0.2%)	0
Melanoma_80	0	0	0	0	0	**125 (100%)**
Melanoma_81	2 (1.5%)	0	**131 (98.5%)**	0	0	0
Melanoma_82	0	0	32 (100%)	0	0	0
Melanoma_84	1 (7.1%)	1 (7.1%)	0	0	1 (7.1%)	**11 (68.7%)**
Melanoma_88	**116 (99.1%)**	0	0	0	0	1 (0.9%)
Melanoma_89	1 (1.0%)	0	0	0	**97 (99.0%)**	0
Melanoma_94	**6 (60.0%)**	1 (10.0%)	2 (20.0%)	0	1 (10.0%)	0

**Table 3:** Dense subgraphs detected by $L_{1}$ analysis.

Subtype	Subgraph	Eigenvector	Subgraph Size	Subgraph Density
Subtype 1	Subg 1	$e_{5}$	20	100%
	Subg 2	$e_{11}$	9	100%
Subtype 2	Subg 1	$e_{62}$	10	100%
	Subg 2	$e_{12}$	13	100%
Subtype 3	Subg 1	$e_{36}$	12	100%
	Subg 2	$e_{23}$	15	100%
Subtype 4	Subg 1	$e_{13}$	13	100%
	Subg 2	$e_{11}$	13	100%
Subtype 5	Subg 1	$e_{26}$	12	100%
	Subg 2	$e_{27}$	9	100%
Subtype 6	Subg 1	$e_{32}$	8	100%
	Subg 2	$e_{9}$	13	100%

**Table 4:** Significant genes and Gene Ontology (GO) analysis of the co-expression networks of different melanoma subtypes.

Subgraph	Gene List	Term Type & Name	*p*-Value
Subtype 1: Subg 1	*UHRF1, TK1, UBE2T, FANCI*,	BP: cell cycle (18)	1.4 × 10${}^{-15}$
	*DTL, TYMS, CENPF, NUSAP1*,	BP: nuclear division (13)	1.2 × 10${}^{-13}$
	*BIRC5, TOP2A, UBE2C, CENPM*,	CC: chromosome (11)	6.4 × 10${}^{-8}$
	*TPX2, CDK1, ANLN, ASPM*,	KEGG: Cell cycle (3)	8.4 × 10${}^{-3}$
	*BUB1, MKI67, PKMYT1, AURKB*		
Subtype 2: Subg 2	*GGH, TK1, TYMS*,	BP: sister chromatid segregation (8)	5.4 × 10${}^{-11}$
	*BUB1, UBE2C, BIRC5, CENPF*,	BP: mitotic cell cycle process (10)	6.5 × 10${}^{-10}$
	*ANLN, NUSAP1, UBE2T*,	BP: chromosome organization (8)	5.2 × 10${}^{-6}$
	*TOP2A, NCAPH, KIFC1*	KEGG: Pyrimidine metabolism (3)	8.5 × 10${}^{-2}$
Subtype 3: Subg 2	*LMNB1, CKAP2, FOXM1, TTK*,	BP: mitotic cell cycle process (13)	6.2 × 10${}^{-15}$
	*NDC80, DEPDC1B, KIF20A*,	BP: cell division (10)	4.2 × 10${}^{-11}$
	*KIF4A, CDKN3, FAM64A, KIF14*,	CC: spindle (9)	8.8 × 10${}^{-11}$
	*RACGAP1, CCNB1, SKA3, KIF20B*	BP: microtubule-based process (9))	3.8 × 10${}^{-9}$
		MF: microtubule binding (5)	1.2 × 10${}^{-5}$
		KEGG:Cell cycle (2)	1.8 × 10${}^{-2}$
Subtype 4: Subg 2	*ORC6, KIF20B, RTKN2, EZH2*,	BP: mitotic cell cycle (9)	5.1 × 10${}^{-8}$
	*CENPW, BRCA2, ARHGAP11B*,	BP: organelle fission (6)	4.6 × 10${}^{-5}$
	*KIAA1524, TIMELESS*,	CC: centrosome (6)	3.7 × 10${}^{-4}$
	*CEP55, PLK4, ESPL1, NEIL3*	BP: DNA metabolic process (5)	4.3 × 10${}^{-3}$
Subtype 5: Subg 2	*CDCA7, MCM4, DSCC1*,	BP: DNA replication (7)	7.2 × 10${}^{-10}$
	*CHAF1A, E2F7, HELLS*,	CC: chromosomal part (7)	8.5 × 10${}^{-7}$
	*GINS2, MCM5, MCM10*	MF: helicase activity (4)	3.6 × 10${}^{-5}$
		BP: cellular macromolecule (9)	6.7 × 10${}^{-5}$
		KEGG: DNA replication (2)	5.2 × 10${}^{-3}$
Subtype 6: Subg 2	*FANCI, TYMS, BIRC5, ASPM*,	BP: mitotic cell cycle (11)	2.5 × 10${}^{-11}$
	*PRC1, CENPF, TK1*,	BP: organelle fission (9)	1.6 × 10${}^{-9}$
	*TOP2A, KIF14, NDC80*,	CC: condensed chromosome (6)	4.4× 10${}^{-7}$
	*HMGB2, MKI67, CDC20*	KEGG: Pyrimidine metabolism (2)	5.7 × 10${}^{-2}$
